# P-207. A review of Imported Fever presentations across two Irish tertiary academic teaching hospitals

**DOI:** 10.1093/ofid/ofaf695.429

**Published:** 2026-01-11

**Authors:** Rhea O’Regan, Caoimhe Patchett, Ellen Sugrue, Catherine Fleming, Christine Kelly

**Affiliations:** Mater Misericordiae University Hosptial, Dublin, Ireland, Dublin, Dublin, Ireland; Mater Misericordiae University Hospital, Dublin, Dublin, Ireland; Galway University Hospital, Dublin, Dublin, Ireland; Galway University Hospital, Dublin, Dublin, Ireland; University College Dublin, Dublin, Dublin, Ireland

## Abstract

**Background:**

Imported fevers are a broad and complex set of presentations to the emergency department without standardised management approaches. Malaria accounts for 22% of all imported fever presentations with viral aetiologies accounting for 45% of presentations. The aim of this audit across two hospital sites was to review clinical data recorded with the aim of establishing a standardised clinical pathway for systematic identification of imported fevers that can be implemented nationally.

**Methods:**

A retrospective audit was conducted of patients across two time periods in large tertiary hospitals with ID services. In one hospital site, imported fever presentations were identified using electronic coding and cross referenced to malaria tests sent across a five-year period. In the second site, they were identified through malaria tests sent over one year.

**Results:**

A total of 73 cases were identified across two hospital sites (26 in one site and 47 in second site) with a documented travel history. Viral haemorrhagic fever assessment occurred in 5(7%) of cases. This was indicated in 22(30% )cases. The most common reason for travel was visiting friends and relatives in 32% (n=26) of cases. Frequent travel locations were Nigeria (n=23, 32%), Malawi (n=4,5%), Uganda (n=4,5%) and Ghana (n=4, 5%). Malaria prophylaxis was taken in11% (n=8) of cases with 5 cases undocumented. Travel vaccination status was recorded in 12(16%) of cases. The most common diagnosis was malaria in 27(37%) of cases. This was followed by 4(5%) invasive bacterial infections, 2(3%) dengue fever, and 2(3%) rickettsioses. An aetiology was not identified in 24(33%) presentations. 66(90%) patients were admitted to hospital. 32(44%) patients received antimicrobials. Additional serology was sent for 34(47%) patients.

**Conclusion:**

People visiting friends and relatives is the most common cohort presenting with imported fever. Malaria was the most common diagnosis, and rates of prophylaxis were low. In terms of clinical assessment, consideration of VHF was poor as was vaccination history. One third of patients did not receive a diagnosis, whilst testing and treatment practices were haphazard.

This study highlights the need for a standardised approach to the assessment and management of imported infections in Irish hospitals.

**Disclosures:**

All Authors: No reported disclosures

